# Endophytic Bacteria *Bacillus subtilis*, Isolated from *Zea mays*, as Potential Biocontrol Agent against *Botrytis cinerea*

**DOI:** 10.3390/biology10060492

**Published:** 2021-06-01

**Authors:** Hernando José Bolivar-Anillo, Victoria E. González-Rodríguez, Jesús M. Cantoral, Darío García-Sánchez, Isidro G. Collado, Carlos Garrido

**Affiliations:** 1 Departamento de Química Orgánica, Facultad de Ciencias, Campus Universitario Río San Pedro s/n, Torre sur, 4 planta, Universidad de Cádiz, 11510 Puerto Real, Spain; hbolivar1@unisimonbolivar.edu.co; 2 Programa de Microbiología, Facultad de Ciencias Básicas y Biomédicas, Universidad Simón Bolívar, Barranquilla 080002, Colombia; 3 Departamento de Biomedicina, Biotecnología y Salud Pública, Laboratorio de Microbiología, Facultad de Ciencias del Mar y Ambientales, Universidad de Cádiz, 11510 Puerto Real, Spain; victoriaeugenia.gonzalez@uca.es (V.E.G.-R.); jesusmanuel.cantoral@uca.es (J.M.C.); dario.garcia@uclouvain.be (D.G.-S.)

**Keywords:** *Botrytis cinerea*, *Bacillus subtilis*, biocontrol, plant promotion, *Zea mays*, *Phaseolus vulgaris*, endophytic bacteria

## Abstract

**Simple Summary:**

Plant–microorganism associations date back more than 400 million years. Plants host microorganisms that establish many different relationships with them, some negative and others very positive for both organisms. A type of this relationship is established with microorganisms that live inside them, known as endophytic microorganisms; they can include bacteria, yeasts, and fungi. In this study, we isolate endophytic bacteria from maize plants, and we characterize them in order to check their potential for being used as biocontrol agents against *Botrytis cinerea*, one of the most important phytopathogenic fungi in the world. The endophytic bacteria showed this antagonistic effect during in vitro assay and also during in vivo assay in *Phaseolus vulgaris*. At the same time, they showed the capacity for promoting growth in *Zea mays* plants.

**Abstract:**

Plant diseases are one of the main factors responsible for food loss in the world, and 20–40% of such loss is caused by pathogenic infections. *Botrytis cinerea* is the most widely studied necrotrophic phytopathogenic fungus. It is responsible for incalculable economic losses due to the large number of host plants affected. Today, *B. cinerea* is controlled mainly by synthetic fungicides whose frequent application increases risk of resistance, thus making them unsustainable in terms of the environment and human health. In the search for new alternatives for the biocontrol of this pathogen, the use of endophytic microorganisms and their metabolites has gained momentum in recent years. In this work, we isolated endophytic bacteria from *Zea mays* cultivated in Colombia. Several strains of *Bacillus subtilis*, isolated and characterized in this work, exhibited growth inhibition against *B. cinerea* of more than 40% in in vitro cultures. These strains were characterized by studying several of their biochemical properties, such as production of lipopeptides, potassium solubilization, proteolytic and amylolytic capacity, production of siderophores, biofilm assays, and so on. We also analyzed: (i) its capacity to promote maize growth (*Zea mays*) in vivo, and (ii) its capacity to biocontrol *B. cinerea* during in vivo infection in plants (*Phaseolus vulgaris*).

## 1. Introduction

Plant disease is one of the main factors responsible for the loss of food in the world. Of all diseases, 20–40% are caused by pathogenic microorganisms, including viruses, bacteria, and fungi [1,2]. *Botrytis cinerea* is considered the second most prevalent plant pathogen worldwide and is responsible for incalculable economic loss owing to its wide spectrum of hosts, multitude of attack modes, high genetic variability, short life cycle, and ability to survive for long periods of time, and to do so as mycelium, conidia, or sclerotia [3]. Today, *B. cinerea* is controlled mainly by synthetic fungicides whose frequent application increases the risk of resistance. In fact, *B. cinerea* is considered a “high risk” pathogen in terms of its resistance to fungicides, which limits the extent to which chemical control can continue to be used [4]. Furthermore, chemical fungicides’ harmful effects on humans, animals, and the environment (air, soil, and water) are well documented [5]. Since 2011, the use of chemical agents to control phytopathogens has declined, mainly in the USA, driven by environmental protection and consumer health regulations [6]. As a result, greater attention has been paid to microorganisms and their metabolites to control plant disease, since these have no negative impact on human or animal health, are environmentally friendly, and, unlike chemical compounds, have no adverse effect on other beneficial microorganisms [1,2]. Of the different microorganisms used in biological control, endophytic microorganisms have attracted the attention of researchers in recent decades [7].

Plant–microorganism associations date back more than 400 million years; while plants are considered as single living organisms, the reality is that each plant is a complex community, considering its participation in various heterospecific associations [8]. Plants host microorganisms that establish mutualistic, commensal, pathogenic, and other sorts of relationships with them [9]. Endophytic microorganisms are an important part of plant microbiota. These microorganisms inhabit plant tissues during part of their life cycle and are not known to cause any disease. In fact, they are generally considered beneficial to promote plant growth [8,9,10]. In addition, they frequently exhibit antagonistic behavior which can be direct (physical contact, hyper-parasitism, predation, and others), indirect (plant defense stimulation, competition for substrates, among others), or mixed antagonism (antibiotics, lytic enzymes, etc.) [11,12]. In addition, endophytes can promote plant growth through mechanisms such as biological nitrogen fixation, phosphate and potassium solubilization, and siderophore production [13,14]. Many endophytes are also able to synthesize one or several phytohormones such as auxins, cytokinins, and gibberellins, having an impact on plant hormonal balance [13,14,15]. Endophytic microorganisms can also indirectly promote growth by acting as antagonists against phytopathogenic agents. They do this by producing antibacterial, antiviral, or antifungal molecules that compete for the niche or by inducing resistance in the plant [15]. As a result of its recent isolation from apparently healthy plant tissue, it was proposed that *B. cinerea* could colonize plants during the course of its life cycle [16,17]. An infection of this sort could become aggressive at later stages (flowering, product storage, among others), with symptoms of infection emerging later [17]. Due to *B. cinerea*’s sometimes endophytic behavior, the use of antagonists with this same mode of action is considered an effective way to control this phytopathogen [16].

Endophytic microorganisms of the *Bacillus* genus are among the most promising studied to date. Several species of this genus have been classified as plant growth promoters and biological control agents, among which *B. subtilis* stands out [18]. The US Food and Drug Administration has granted *B. subtilis* “generally regarded as safe” status. Not being considered a pathogen gives it advantages for use as a biocontrol agent [19]. The *Bacillus* genus is also capable of producing endospores that are tolerant of heat, desiccation, UV irradiation, and organic solvents, a clear advantage in the formulation and storage of biocontrol products [7,10,20,21]. Hence, there are many fungal disease biocontrol products on the market that contain *B. subtilis* strains as one of their active ingredients (e.g., Serenade^®^ Max and Companion) [22]. Different strains of *B. subtilis* behave in different ways and have varying capacities, making it is necessary to select an efficient and stable strain for use as a biocontrol agent. This species can produce a wide range of antimicrobial compounds, among which cyclic lipopeptides (iturine, fengycin, and surfactins), with a broad antifungal spectrum, stand out [21,23,24].

This paper describes the isolation of endophytic bacteria from maize plants (*Zea mays*) cultivated in Colombia, evaluates the use of these endophytic strains to control *B. cinerea* strain B05.10, during in vitro and also during in vivo assays in *Phaseolus vulgaris*, and studies their ability to promote plant growth.

## 2. Materials and Methods

### 2.1. Strains and Culture Conditions

Seven strains were used: wild type *Botrytis cinerea* B05.10, one strain of *Pseudomonas aeruginosa,* and five *Bacillus subtilis* isolated from *Zea mays*, identified in this study (Table 1). Potato Dextrose Agar medium (PDA) (Condalab S.A., Madrid, Spain) was used for routine fungal cultures and Tryptone Glucose Yeast Extract Agar (YGA) (Oxoid) to isolate bacteria from maize plants. Cultures were incubated at 25 °C under 12 h-daylight/12 h-darkness.

### 2.2. Isolation of Endophytic Microorganisms from Maize

Healthy maize plants (*Zea mays*) grown in Colombia’s Department of Atlántico were sampled from various locations with different geographical features. Freshly collected samples were brought to the laboratory in sterile packaging and immediately processed. Maize stems were washed with sterile distilled water (SDW) to remove soil and other unwanted particles and then sterilized with 80% ethanol for 2 min, followed by a wash with 4% sodium hypochlorite for 5 min and concluding with 8 successive washes with SDW [26]. Surface sterilization was confirmed by the absence of any microbial growth on YGA agar plates inoculated with aliquots of the final rinse water. Superficial stem bark was then removed using a sterile scalpel, and internal tissue segments were macerated with 1 mL of sterile 0.9% NaCl in a sterile mortar. The macerate dilution and tissue segments were placed on YGA plates incubated at 25 °C for 72 h [26,27]. Bacterial isolates were selected based on colony appearance and streaked on YGA plates until purity level was achieved. The isolates purified were maintained on Luria Bertani (LB)-agar plates (Condalab S.A.) at 25 °C and cells were stored in 30% (*v*/*v*) glycerol at −80 °C for later studies [28].

### 2.3. Antagonistic Activity Assay against Botrytis cinerea

To evaluate and select the bacterial isolates with antagonistic effects against *B. cinerea* B05.10, the isolated strains were inoculated on PDA with five-millimeter mycelia discs of seven-days-old culture of *B. cinerea* (1 cm apart). Antagonistic assays were incubated at 25 °C for seven days. All bacterial isolates were evaluated in three independent replicates. The antagonistic effect was calculated as described by Tenorio-Salgado et al. (2013) [29].

$$\mathrm{Radial}\;\mathrm{Inhibition}\;\left(\%\right):\;\left(\frac{Rc-R1}{Rc}\right)100$$

*Rc* is the mean value of fungus radius with no bacteria. *R*1 is fungus radius in the presence of the antagonistic bacteria. Isolates exhibiting over 40% radial inhibition were selected for further analysis.

### 2.4. Identification of Bacteria

#### 2.4.1. Molecular Identification

Bacterial genomic DNA was isolated as described by González-Rodríguez et al. (2016) [30]. Two pairs of primers were used for partial 16S-rRNA gene amplification prior to sequencing: primers *16SF-16SR,* from Scarpellini et al. (2004) [31], and *Bac_Fwd-Bac_Rev1,* designed as part of this study using the DNASTAR^®^ Lasergene package (DNASTAR, Inc., Madison, WI, USA) (Table 2). PCR amplifications were performed in a SimpliAmp Thermal Cycler (Applied Biosystems, Foster City, CA, USA) as follows: a total volume of 50 μL containing 1× buffer, 1.5 mM MgCl_2_, 0.2 mM dNTP, 0.2 μM of each primer, 2.0 U of Go-Taq^®^ DNA Polymerase (Promega), and 0.5 μg of genomic DNA. Cycling conditions were as follows: 95 °C for 5 min, 35 cycles of 95 °C for 1 min, 52 °C for 1 min, and 72 °C for 2 min, and a final extension step at 72 °C for 10 min. Gel electrophoresis separations were performed using standard procedures [32], and products were purified using the GeneJET PCR Purification Kit (Thermo Scientific).

Purified products were quantified using a Thermo Scientific™ (Waltham, MA, USA) NanoDrop 2000c spectrophotometer, diluted to 30 ng μL^−1^ and sent to the Genomic Unit of the University of Cordoba (Spain) for sequencing. Both strands of PCR products were sequenced. Sequences were assembled, and complementary strands were compared using the Basic Local Alignment Search Tool (BLAST) with the nucleotide database from the National Centre for Biotechnology Information (NCBI). Sequences were aligned using the ClustalW algorithm, and a neighbour-joining phylogenetic analysis was conducted using MegAlign from the DNASTAR^®^ Lasergene package (DNASTAR, Inc., Madison, MI, USA). To study the phylogenetic relationship of our isolates, another 27 sequences of related *Bacillus* species and three additional sequences of related genera, *Anoxybacillus*, *Geobacillus*, and *Saccharococcus,* were downloaded from the GenBank database and included in the phylogenetic tree.

#### 2.4.2. Specific PCR for *Bacillus subtilis*

To ensure molecular identification, the set of specific primers *Bsub5F-Bsub3R* previously described by Wattiiau et al. (2001) [33] were used to amplify the internal fragment of the ‘*Bacillus subtilis* group’ of the 16S-rRNA gene. PCR amplifications were conducted in a SimpliAmp Thermal Cycler (Applied Biosystems) as described above, and cycling conditions were as follows: 95 °C for 5 min, 35 cycles of 95 °C for 1 min, 63 °C for 1 min, 72 °C for 2 min, and a final extension step at 72 °C for 10 min [33].

### 2.5. Phenotypical Characterization of B. subtilis Isolates

#### 2.5.1. Discriminatory Carbon Source Assimilation

Differences in carbon sources were analysed with the API-50CH, according to the manufacturer’s recommendations (BioMérieux, Marcy l’Etoile, France). Results were obtained after 6 days of incubation at 25 °C. All assays were done in triplicate [35].

#### 2.5.2. Detection of Genes Involved in the Synthesis of Lipopeptides and Quantification in *Bacillus subtilis*

The genes from the isolates involved in lipopeptide pathways were studied. A total of six genes were detected by conventional PCR using primers as described by Mora et al. (2011) [34]: *ituC*, *fenD*, *bacA*, *srfAA*, *spaS*, and *bmyB* genes (Table 2). The PCR was run at a total volume of 50 μL containing 1× buffer, 1.5 mM MgCl_2_, 0.2 mM dNTP, 0.2 μM of each primer, 2.0 U of Go-Taq^®^ DNA Polymerase (Promega), and 0.5 μg of genomic DNA. The cycling conditions were as follows: 95 °C for 5 min, 40 cycles of 94 °C for 1 min, annealing temperature for 1 min, and 72 °C for 1 min. A final extension step at 72 °C for 10 min was followed by a 4 °C soak. The annealing temperature was set at 58 °C for *fenD*, *ituC*, *srfAA*, *bacA* and *spaS*, and to 55 °C for *bmyB* [34]. PCR products were separated by gel electrophoresis using standard procedures [32].

Lipopeptides produced by *B. subtilis* were quantified following the protocol optimized by Mukherjee et al. (2009) and Meng et al. (2016) [36,37] using a turbidimetric method. Briefly, a single colony was placed into 96 deep-well plastic plates containing 1.5 mL liquid culture media (g·L^−1^: sucrose 20; NH_4_Cl_3_; KH_2_PO_4_ 3.5; Na_2_HPO_4_ 5 and yeast extract 0.5) and supplemented with a micro salt solution (mg·L^−1^: FeSO_4_·7H_2_O 0.85; ZnSO_4_·7H_2_O 0.4; MgSO_4_·7H_2_O 2; MnSO_4_·H_2_O 0.2). Multi-well plates were incubated for 48 h at 20 °C and with rotation 120 rpm. Then, cells were separated by centrifugation and 100 μL cell-free supernatant were mixed with 50 μL 10% trichloroacetic acid and placed on a new multi-well plate. After 30 min of incubation at room temperature (RT), turbidity was checked by Microplate Reader (MultiSkanTM FC—Thermo Scientific, Waltham, MA, USA) at 600 nm. As negative controls, SDW was used instead of the 10% trichloroacetic acid. All assays were done in triplicate.

#### 2.5.3. Indole Acetic Acid Production (IAA)

The production of IAA by *B. subtilis* was studied using the protocol described by Glickmann and Desseaux (1995) [38]. Single bacterial colonies were inoculated in 10 mL of King-B medium (Condalab S.A.) and incubated at 25 °C for 48 h and with rotation 180 rpm. Then, cells were separated by centrifugation at 10,000 rpm. Equal volumes of Salkowsky’s reagent and supernatant were mixed (1:1) and incubated in the dark at RT for 30 min in a spectrophotometer cuvette. Then, absorbance was measured at 530 nm. IAA concentration in each sample was determined from the standard curve of IAA (mean of three values) within the range of 0 to 200 µg.mL^−1^ [38]. All assays were done in triplicate.

#### 2.5.4. Phosphate Solubilization

This assay was performed according to Shahid et al. (2015) [28]. Phosphate solubilization by *B. subtilis* isolates was quantified using Pikovskaya’s solid medium (g·L^−1^: yeast extract 0.5; glucose 10; Ca_3_(PO_4_)_2_ 5.0; (NH_4_)_2_SO_4_ 0.5; KCl 0.2; MgSO_4_ 0.1; MnSO_4_ 0.001; FeSO_4_ 0.0001; and agar 18; pH: 7.0–7.4). Bacterial colonies were inoculated, then incubated for 10 days at 25 °C. The formation of a clear halo around the colony was considered positive for phosphate solubilization. All assays were done in triplicate.

#### 2.5.5. Potassium Solubilization

Colonies were inoculated in Aleksandrov medium described by Zhang and Kong (2004) (g·L^−1^: glucose 5, MgSO_4_·7H_2_O 0.5; FeCl_3_ 0.005; CaCO_3_ 0.1; CaPO_4_ 2; and KAlSi_3_O_8_ 2; pH: 7.0–7.5) [39]. The formation of a clear halo around the colony after 72 h of incubation at 25 °C was considered positive [39].

#### 2.5.6. Growth in Nitrogen-Free Medium

To characterize the ability of the strains to use atmospheric nitrogen for growth, JMV semisolid medium (g·L^−1^: mannitol 5.0; K_2_HPO_4_ 0.6; KH_2_PO_4_ 1.8; MgSO_4_·7H_2_O 0.2; NaCl 0.1; CaCl_2_·2H_2_O 0.02; yeast extract 0.05; and agar 1.6; pH 5.5–5.7) was inoculated with bacterial colonies and incubated at 25 °C for 7 days as described by Reis et al. (2004) [35] and Baldani et al. (2014) [40]. Bacterial growth indicated a positive result in this test. All assays were done in triplicate [35,40].

#### 2.5.7. Proteolytic Activity

Skimmed milk medium (10%) was used to calculate the proteolytic activity of *B. subtilis* following the protocol optimized by Castro et al. (2014) (g·L^−1^: tryptone 5; yeast extract 2.5; glucose 1; NaCl 2.5; and agar 18; pH 7.0—add 100 mL skimmed milk after sterilization) [41]. Single colonies were inoculated in solid medium and incubated at 25 °C for 72 h. The presence of halos around the colonies was considered positive. *Pseudomonas aeruginosa* strain from our laboratory collection was used as positive control for proteolytic activity. All assays were done in triplicate [41].

#### 2.5.8. Amylolytic Activity

The capacity to transform starch into sugar through the action of enzymes was characterized by inoculating a single colony of *B. subtilis* on starch agar plates (5% tryptone soya agar (TSA) (Oxoid) medium supplemented with 1% soluble starch) and incubated for 72 h at 25 °C. Then, 5 mL of a 1% iodine solution was added to reveal the result. The presence of clear halos around the colonies was considered positive. All assays were done in triplicate [41].

#### 2.5.9. Siderophore Detection

The protocol described by Alexander and Zuberer (1991) [42] was selected for siderophore detection in *B. subtilis*. Strains were inoculated in King-B medium (Condalab S.A., Madrid, Spain), and incubated at 25 °C, with rotation 120 rpm for 7 days. Then, culture medium was centrifuged at 10,000 rpm and 100 μL of supernatant was mixed with an equal volume of 2 mM chrome azurol S solution. The mixture was incubated at RT for 30 min. Change in colour to yellow-orange indicated the production of hydroximate-type siderophores, and to purple indicated the production of catechol-type siderphores [42]. *Pseudomonas aeruginosa* strain from our laboratory collection was used as positive control for siderophore detection.

#### 2.5.10. Biofilm Assays

The ability of bacteria to produce biofilm can be estimated by using solid-surface-associated biofilm formation with the crystal violet (CV) staining method described by Almoneafy et al. (2014) [43] and Merritt et al. (2015) [44]. Bacterial strains were cultured in LB medium for 18 h at 25 °C. Fresh cultures were diluted to 0.3 (OD600–10^7^ CFU/mL). Then, 5 μL was added to 195 μL of LB medium in 96-well plates and incubated at 25 °C for 24 h. The culture medium was removed from the wells, and each well was gently rinsed with SDW. Then, 150 μL of 1% CV was added, and the culture was incubated at RT for 30 min. Each well was washed 2 more times with SDW. The CV attached to the biofilm was solubilized in 150 mL of 33% acetic acid and quantified by measuring its absorbance at 570 nm using a microplate reader (MultiSkanTM FC—Thermo Scientific) [43,44] *Pseudomonas aeruginosa* strain from our laboratory collection was used as positive control for biofilm production.

### 2.6. Botryane Production in Antagonist Test

A study of the production of botryanes (botrydial + dihydrobotrydial) was conducted during the common growth of the B05.10 strain and antagonistic *B. subtilis*. The study was done in triplicate on PDA plates where fungus and antagonistic bacteria were inoculated at opposite ends of the plates and incubated at 25 °C for 7 days. Then, (12) PDA plugs containing the mycelia (1 cm diameter/plug) were taken from two different sites: (i) from the fungus–bacteria interaction zone, and (ii) from the non-interaction part of the fungus furthest from the bacteria.

Botryanes were extracted following the protocol optimized by Izquierdo-Bueno et al. (2018) [45]. Briefly, twelve PDA plugs were extracted with ethyl acetate (3 × 300 mL) using an ultrasonic bath for 30 min. The ethyl acetate organic extract was dried over Na_2_SO_4_, concentrated to dryness, and then separated in a chromatography column (silica gel) eluted with ethyl acetate-hexane (40:60). The isolated botryanes were identified by thin-layer chromatography and characterized by ^1^H-NMR. The production of botryanes was expressed in micrograms of botryanes per millilitre of medium (μg botryanes·mL^−1^).

### 2.7. Evaluation of Plant Growth Promotion by Endophytic Strains under Greenhouse Conditions

#### 2.7.1. Bacteria Encapsulation in Alginate Beads

*B. subtilis* was cultured in LB broth and incubated at 25 °C, with rotation of 120 rpm for 24 h. Bacteria were recovered by centrifugation and resuspended in 0.9% NaCl until final OD600 of 1.8. Alginate beads were obtained by preparing a 1.25% sodium alginate solution, constantly stirred at 120 °C until a homogeneous solution was obtained. The composition also included glycerin (as an osmoprotector) and sucrose to better dissolve the alginate, and as an additional source of nutrition. The sodium alginate mixture was cooled to 50 °C, and the bacteria in suspension were added and mixed slowly. With the aid of a positive pressure pump, the alginate solution mixture containing the bacterial cells was added dropwise to a 2% CaCl_2_ solution while maintaining constant agitation for 45 min until stabilization of the beads. Beads were then removed from the CaCl_2_ solution and washed in 0.9% sterile NaCl. Beads were immediately used for bacterial counting and plant growth promotion tests [46].

#### 2.7.2. Bacterial Viability Evaluation after Encapsulation

To study the viability of *B. subtilis* during encapsulation, 1 g of alginate beads were disaggregated by adding a 2% sodium citrate solution. Then, serial dilution was performed using 0.9% NaCl and LB agar medium. Plates were incubated for 3 days at 25 °C, after which CFU bacteria·g^−1^ of the bead was calculated [46].

#### 2.7.3. Maize Seed Inoculation and Growth

Maize seeds were superficially sterilized by thorough washing with SDW, and then washed with 80% ethanol under stirring for 20 min and at 5% under agitation for 10 min. Ethanol was drained, and 1.3% NaOCl was added under stirring for 30 min. Finally, 5 washes were performed with SDW. To verify surface sterility, 5 seeds per treatment were placed on LB agar and incubated for 10 days at 25 °C [47,48].

Disinfected maize seeds were grown together with the *Bacillus subtilis* alginate beads to enhance their endophytic relationship as described below. Greenhouse pot experiments were conducted in a sterile mix of vermiculite, sand, and clay (1:1:1). A single seed per pot was sown on 2 g of alginate beads containing the bacteria. Negative controls followed the same procedure but with alginate beads that did not contain bacteria. Pots were irrigated with sterile 4-fold-diluted Hoagland solution. Plants were grown for 5 weeks in a growth chamber with a 17/7 h photoperiod, at a temperature of 27 ± 2 °C and 60 ± 2% humidity. Plant fresh weight, number of leaves, and stem and root size was calculated after 5 weeks of growth [47,49].

#### 2.7.4. Isolation and Molecular Detection of *B. subtilis* from Greenhouse-Inoculated Maize Plant

After evaluation of plant growth promotion, *B. subtilis* was isolated and identified from the stems of inoculated maize plants. For bacterial isolation, stems of inoculated plants were sampled, and their surface externally sterilized, as explained previously. Stems of negative control plants were also included in this assay. Internal tissue segments were macerated with 1 mL of sterile 0.9% NaCl in a sterile mortar. The macerate dilution and tissue segments were placed on YGA and incubated at 25 °C for 72 h. Bacterial colonies were collected and used for the molecular identification of the *B. subtilis* species. In order to identify *B. subtilis*, a double experiment was carried out using: (i) the isolated bacteria and (ii) surface-sterilized stems of inoculated plants to detect and identify *B. subtilis* directly from plant material. Total DNA was extracted from both sample types in independent experiments using the protocol described by González-Rodríguez et al. (2016) [30]. Then, species-specific primers *Bsub5F-Bsub3R* previously described by Wattiiau et al. (2001) [33] were used to amplify the internal fragment of the ‘*Bacillus subtilis* group’ of 16S-rRNA gene as described above.

### 2.8. Evaluation of Antagonistic Effect of B. subtilis during B. cinerea Infection on Phaseolus vulgaris

To evaluate the antagonistic effects against *B. cinerea* B05.10, infection assays were carried out on *Phaseolus vulgaris* (French bean), which is a classical sensible hosts of *B. cinerea* for these studies.

#### 2.8.1. Bean Seed Inoculation with *B. subtilis* and Molecular Detection in Plant

To study the viability of *B. subtilis* as endophytic bacteria in *P. vulgaris,* bacteria cells were encapsulated in alginate beads, and bean seeds were inoculated, as described above (Section 2.7.3). Plants were grown for 12 days in a growth chamber with a 17/7 h photoperiod, at a temperature of 24 ± 2 °C and 60 ± 2% humidity, until primary leaves appeared. After, *B. subtilis* was isolated and identified from the leaves of inoculated beans plants as described above (Section 2.7.4).

#### 2.8.2. Infection Assays with *B. cinerea*

Infection assays were performed on primary leaves of *P. vulgaris* containing *B. subtilis* inside them, and plants without bacteria as control. Leaves of living plants were inoculated with 4-μL droplets of conidial suspensions (2 × 10^5^ conidia/mL) of *B. cinerea* B05.10 [30]. Infected plants were grown for 7 days in a growth chamber with a 17/7 h photoperiod, at a temperature of 24 ± 2 °C and 60 ± 2% humidity.

## 3. Results

### 3.1. Isolation of Endophytic Microorganisms from Maize and Antagonistic Activity Assay against Botrytis cinerea

**Figure 1:**
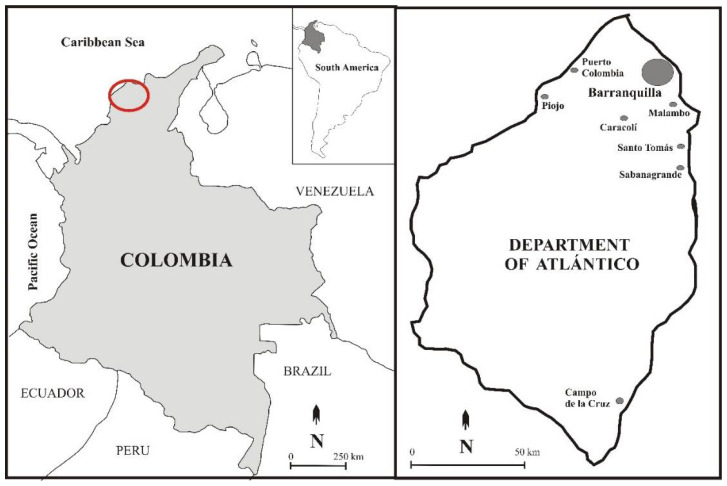
Department of Atlántico region in Colombia. On the left, the Colombia map with a red mark of sample region. On the right, the Department of Atlántico with the name sampling areas/towns.

A total of 40 adult maize plants were sampled from 7 different parts of Colombia’s Department of Atlántico featuring different types of soil and climate (Figure 1). The surface of plant stems was sterilized, and endophytic microorganisms isolated, using the protocol described above. A total of 75 bacteria were isolated from the plant samples.

**Figure 2:**
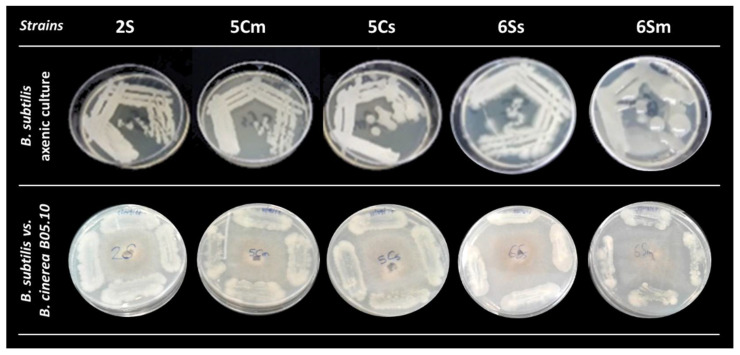
*Bacillus subtilis* strains in axenic culture (**top**-line); *B. subtilis* vs. *B. cinerea* during antagonistic test (**bottom**-line).

All isolates were purified, and their in vitro antagonistic effects against *B. cinerea* B05.10 were studied using the equation established by Tenorio-Salgado et al. (2013) [29]. Twenty-two isolates exhibited an antagonistic effect against *B. cinerea*, but only the five that exceeded the 40% inhibition threshold were selected. These isolates were named 2S, 5Cs, 5Cm, 6Ss, and 6Sm (S:Sabanagrande, C:Campo de la Cruz) (Table 1, Figure 1 and Figure 2 and Supplementary File S1).

### 3.2. Molecular Identification of Endophytic Strains

Bacterial genomic DNA of the five selected isolates was extracted, and two pairs of primers (*16SF-16SR* and *Bac_Fwd-Bac_Rev1*, Table 2) were used to amplify a fragment of 16S rRNA gene. PCR products were purified and sent for sequencing. Nucleotide sequences were deposited in GenBank (http://www.ncbi.nlm.nih.gov/Genbank/ (accessed on 31 May 2021)); accession numbers are shown in Table 1. The sequences were compared with the Nucleotide database using BLAST, and all isolates were identified as *Bacillus subtilis* with a percent identity exceeding 99%.

**Figure 3:**
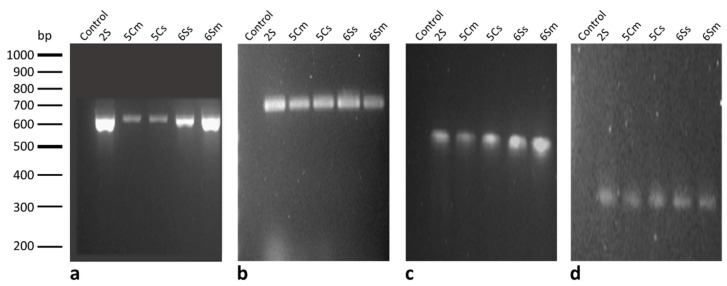
Gel electrophoresis. DNA molecular market: GeneRuler-100bp-Plus. Lines show negative controls (Control) and *B. subtilis* strains (2S, 5Cm, 5Cs, 6Ss, 6Sm). (**a**) PCR products of the internal fragment of the *‘Bacillus subtilis* group’ of 16S-rRNA gene using the species-specific primers *Bsub5F-Bsub3R* (600 bp). (**b**) PCR products of *srfAA* gene (surfactin) (700 bp). (**c**) PCR products of *bacA* gene (bacylisin) (approx. 500 bp). (**d**) PCR products of *bmyB* gene (bacillomycin) (approx. 300 bp).

To ensure the molecular identification done by sequencing, the species-specific primers *Bsub5F-Bsub3R* were used to amplify the internal fragment of the ‘*Bacillus subtilis* group’ of the 16S-rRNA gene. The five isolates amplified a PCR product of 600 bp (Figure 3A). DNA from *P. aeruginosa* strain was included as negative controls. These results confirmed identification of analyzed bacteria.

**Figure 4:**
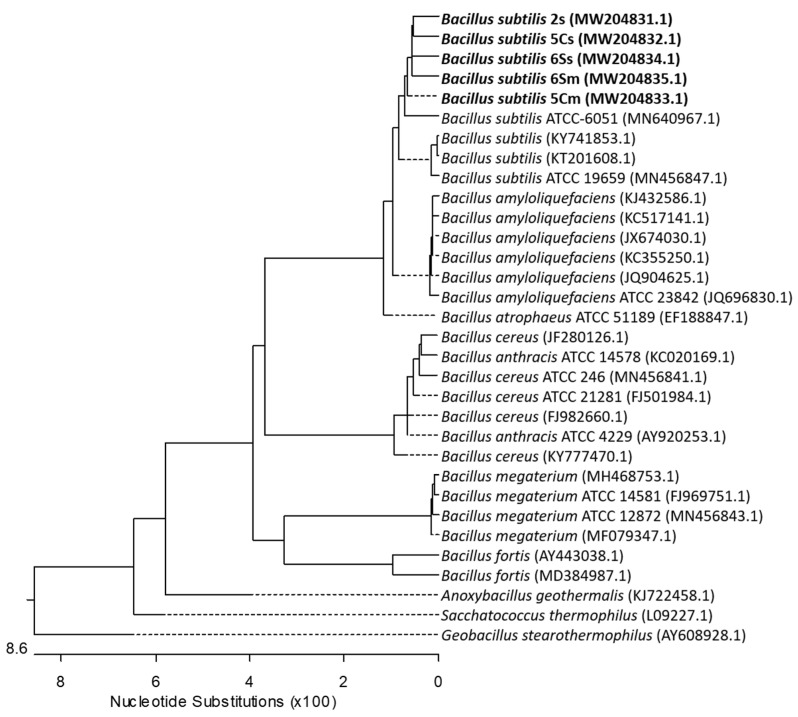
Neighbor-joining tree derived from 16S-rRNA gene sequences of sequenced isolates and published sequences. The length of each pair of branches represents the distance between sequence pairs. A dotted line on the tree indicates a negative branch length; the bar indicates the number of nucleotide substitutions.

A neighbor-joining phylogenetic analysis was conducted using the Kimura two-parameter model and a bootstrap test of 5000 runs (MegAlign, DNASTAR^®^ Lasergene package). Twenty-seven sequences of related bacterial species/genus were downloaded from the GenBank database and included in the analysis. The phylogenetic tree showed a cluster with *B. subtilis* isolated in this study, clearly separated from other *Bacillus* species and from other genus of related bacteria from the family *Bacillaceae* (Figure 4).

### 3.3. Phenotypical Characterization of B. subtilis Isolates

The isolated strains of *Bacillus subtilis* were phenotypically characterized by studying characteristics that could be interesting for plant colonization, its growth as an endophyte, and its potential as a biocontrol agent and plant growth promotor. These tests included morphological and biochemical characteristics, among others. All isolates exhibited the typical rod morphology, positive Gram stain, positive oxidase and catalase activity, and bacterial motility. Differences in carbon source use were analysed using an API-50CH (BioMérieux). Table 3 shows that the isolates exhibited similar capacities to assimilate a large number of carbon sources except erythrose, D-arabinose, D-galactose and D-lactose.

The strains were also screened for their capacity to produce indole acetic acid, solubilize phosphate and potassium, grow in a nitrogen-free medium, and for proteolytic activity, amylolytic activity, siderophore detection, and biofilm formation. All these characteristics are important when analyzing the potential of these *B. subtilis* isolates to promote the growth and development of maize plants (Table 4).

Strains 5Cs and 5Cm exhibited the highest IAA production values, with 3.7 and 2.6 μg·mL^−1^, respectively. All isolates were able to grow in a nitrogen-free medium and exhibited proteolytic and amylolytic activity but no phosphate or potassium solubilization. Siderophores were produced by all, but none produced biofilm.

To complete the phenotypical characterization, we determined whether the genes involved in lipopeptide pathways were present in the isolates. A total of six genes were studied by PCR using the primers listed in Table 2, *ituC*, *fend*, *bacA*, *srfAA*, *spaS*, and *bmyB* genes [34], the genes involved in the biosynthesis of iturin, fengicin, bacylisin, surfactin, subtilin, and bacillomycin, respectively. Among the isolates, only genes *bacA*, *srfAA*, and *bmyB* were detected by PCR, suggesting that the *B. subtilis* strains studied were only able to produce bacylisin, surfactin, and bacillomycin lipopeptides (Figure 3B–D). Total lipopeptide production was quantified using a turbidimetric method optimized by Mukherjee et al. (2009) and Meng et al. (2016) [36,37]. The results presented in Table 4 show that all *B. subtilis* strains produce a similar total amount of lipopetides, that is., approximately 1 mg·mL^−1^, 6Sm being the strain that produced the highest amount, attaining a value of 1.24 mg·mL^−1^.

### 3.4. Botryane Production in Antagonist Tests

**Figure 5:**
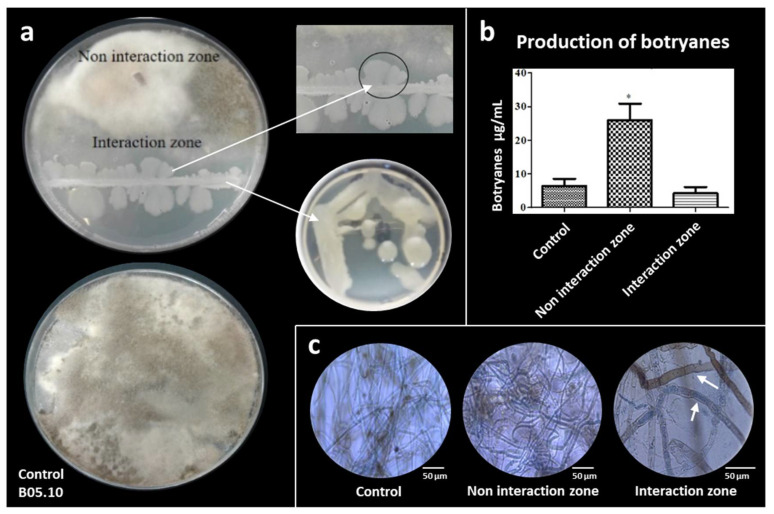
Co-culture of the *B. subtilis* 6Sm and *B. cinerea* B0510. (**a**) Interaction zone described as “contact inhibition”. Image shows changes in the morphology of the colony formed by *B. subtilis* under co-culture (black circle). Small Petri dish shows the growth of *B. subtilis* in axenic culture. (**b**) Production of botryanes from non-interaction zone and interaction zone, in comparison with *B. cinerea* B05.10 control. (**c**) Microscopical characteristics of *B. cinerea* B05.10 hyphae from control, non-interaction, and interaction zone. White arrows point to granulations and macrosiphonate hyphaes.

*B. cinerea* B0510 and *B. subtilis* 6Sm were inoculated on the same PDA plates. During the co-culture, strains came into contact, but with no clear inhibition area (Figure 5A). This type of interaction was described by Bertrand et al. (2013) [50] as “contact inhibition”. Cell mobility was observed in the growth morphology of the *B. subtilis* strain. However, under axenic conditions, this strain has a mucoid phenotype and no apparent mobility (Figure 5A). Biomass of the bacteria, growing in co-culture with *B. cinerea*, was taken, and PCR analysis showed that the bacteria was *B. subtilis*. All co-culture tests were performed with three replicates in three independent assays.

Botryane production by *B. cinerea* during co-cultivation with *B. subtilis* 6Sm was also studied. In the co-culture of *B. cinerea* vs. *B. subtilis*, we observed that the dry fraction weight yielded a value of 26 µg·mL^−1^ and 4.2 µg·mL^−1^, respectively, for the non-interaction and interaction zones, and 6.4 µg·mL^−1^ in the control (Figure 5B). Results show a statistically significant (*p* < 0.05) increase in botryane production in the non-interaction zone. In contrast, in the interaction zone, a decrease in botryane production was observed when compared with normal production by *B. cinerea* under axenic culture conditions, used as the control (Figure 5B). No changes were observed in the morphology of *B. cinerea* hyphae in the non-interaction zone, while hyphae with macrosiphonated and granular cytoplasm were observed in the interaction zone (Figure 5C).

### 3.5. Evaluation of Plant Growth Promotion by Endophytic Strains under Greenhouse Conditions

#### 3.5.1. Bacteria Encapsulation in Alginate Beads

**Figure 6:**
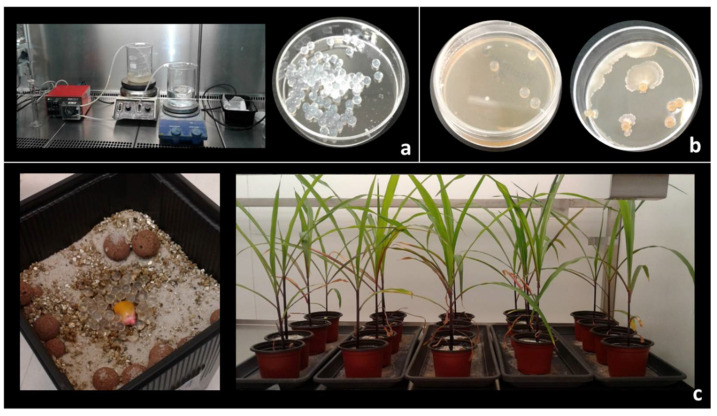
(**a**) Bacteria encapsulation in alginate beads. (**b**) Alginate beads cultivated in LB medium. Bacteria grows outside of spheres. (**c**) Maize seeds inoculated with *B. subtilis* encapsulated in alginate beads. Maize plants growing in the greenhouse.

*B. subtilis* 6Sm was cultured in LB broth, and fresh cells were encapsulated in alginate beads to inoculate maize seeds. Bacteria viability had previously been tested by recovering 1 g of alginate beads and disaggregating them by adding a 2% sodium citrate solution (Figure 6A). Viability counting showed values between 10^6^–10^7^ CFU-bacteria·g^−1^ of bead. Alginate beads were also cultivated in LB solid medium where bacteria were able to grow outside the beads (Figure 6B). Alginate beads obtained using SDW instead of bacterial culture were used as negative control. No growth was observed when the control beads were added to the LB solid medium (Figure 6B).

#### 3.5.2. Evaluation of Plant Growth Promotion

Maize seeds were superficially sterilized and then grown alongside *B. subtilis* alginate beads to enhance the plant–bacteria endophytic relationship. Greenhouse pot experiments were performed using a total of 60 plants: 10 plants inoculated with bacteria and 10 negative control plants using alginate beads obtained with SDW rather than bacteria culture, in three independent assays (Figure 6C).

**Figure 7:**
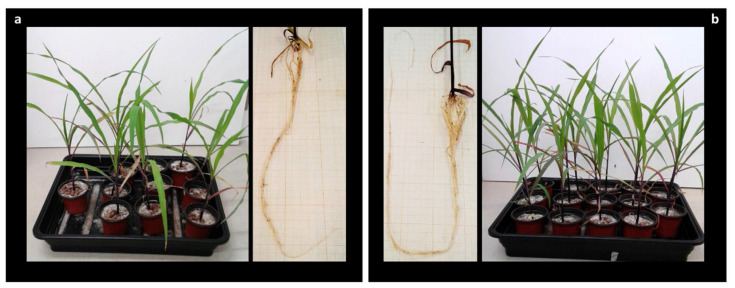
Five-week-old plants of maize. (**a**) Plant growth with alginate beads without bacteria. (**b**) Plant growth with alginate beads containing *B. subtilis*. The roots of the plants inoculated with *B. subtilis* were statistically significantly longer and more branched than the root system of the control plants.

After 5 weeks (17/7 h photoperiod, at a temperature of 27 ± 2 °C and 60 ± 2% humidity), plants were harvested and roots washed with SDW. Then, fresh weight, number of leaves, and stem and root size were measured, and control and inoculated plants compared. Plants looked healthy, showing no signs of disease (Figure 7). Similar results between control and inoculated plants were observed in the aerial parts (number of leaves and stem size) (Table 5) and wet weight. However, significant differences were observed in the root system. The plants inoculated with *B. subtilis* had a significantly larger and more branched root system than the control plants (Table 5 and Figure 7). These same results were obtained in the three independent tests of all plants.

#### 3.5.3. Isolation and Molecular Detection of *B. subtilis* from Greenhouse Inoculated Maize Plants

*B. subtilis* was re-isolated and identified from the stems of plants inoculated with alginate beads using the same protocol described in Section 2.7.4 of Material and Methods. After 72 h of incubation, colonies of bacteria had grown on YGA plates. DNA was extracted from re-isolated colonies and from surface-sterilized stems of inoculated plants (including negative plant controls). Species-specific PCR was used to amplify the internal fragment of the 16S rRNA ‘*Bacillus subtilis* group’. *B. subtilis* was identified by PCR using the total DNA samples from plants inoculated with the microorganisms, while negative amplification results were obtained using total DNA from plants grown using alginate beads with no bacteria. Positive amplifications were also obtained from DNA samples of single bacteria colonies re-isolated from plants in this assay. These results show that *B. subtilis* can establish a symbiosis with maize plants through the inoculation protocol used, and suggest that the differences observed in plant growth is due to the presence of these endophytic microorganisms.

### 3.6. Evaluation of Antagonistic Effect of B. subtilis during B. cinerea Infection on Phaseolus vulgaris

#### 3.6.1. Bean Seed Inoculation with *B. subtilis* and Molecular Detection in Plant

**Figure 8:**
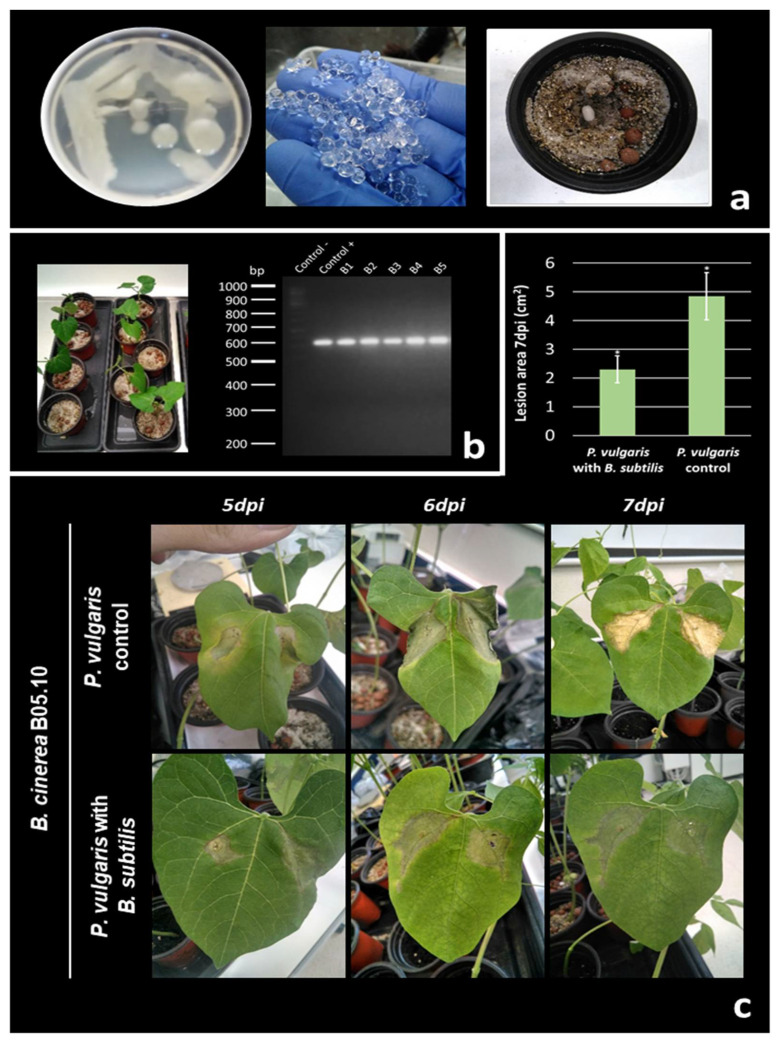
(**a**) Bacteria encapsulation in alginate beads; bean seeds inoculated with *B. subtilis* encapsulated in alginate beads. (**b**) Bean plants growing in the greenhouse; products amplified by PCR with primers *Bsub5F-Bsub3R* (600 bp) on the template of DNA samples extracted from bean plants inoculated with *B. subtilis* (B1-B5) and DNA of *B. subtilis* and *P. aeruginosa* as control positive and negative, respectively. DNA molecular market: GeneRul-er-100bp-Plus. (**c**) The graph shows the mean area of the lesions at 7 days post-inoculation (dpi) (* *p* ˂ 0.05). Images show the course of infection in plant control in comparison with a plant germinated previously with *B. subtilis* along 5-, 6-, and 7-dpi (representative images of the lesions are shown on different leaves).

*B. subtilis* 6Sm was cultured in LB broth and fresh cells were encapsulated in alginate beads, and viability had previously been tested (Section 3.5.1.) (Figure 6A,B and Figure 8A). *P. vulgaris* plants inoculated with *B. subtilis* grew in parallel with control plants. Primary leaves were obtained at 10–12 days under growth chamber condition in both types of plants (Figure 8A). After, *B. subtilis* was re-isolated and identified from the leaves of inoculated plants as described above (Section 2.7.4). Species-specific PCR was used to amplify the internal fragment of the 16S rRNA gene ‘*Bacillus subtilis* group’. Positive amplifications were obtained from DNA samples of single bacteria colonies re-isolated, and also using the template of DNA samples from inoculated plants (Figure 8B). *B. subtilis* cells were not isolated from control plants, and no PCR amplifications were obtained using on the template of DNA from control plants. These results show that *B. subtilis* can also establish a symbiosis with beans plants through the inoculation protocol used. It suggests that the endophytic symbiosis relationship of this strain was not host-specific for the plants under the experimental condition studied.

#### 3.6.2. Infection Assays with *B. cinerea*

To study the infection process, primary leaves of young *P. vulgaris* plants (containing *B. subtilis* inside them, and plants without bacteria as control) were inoculated with conidial suspensions of *B. cinerea* B05.10 and were monitored during 7 days (Figure 8C). Greenhouse pot experiments were performed using a total of 18 plants: 3 plants inoculated with bacteria and 3 control plants, in three independent assays. Results revealed differences between plants containing bacteria inside and control plants: primary lesions became visible after three days of incubation for all plants. Leaves of control plants were clearly deteriorated by *B. cinerea,* showing symptoms of chlorosis. In contrast, leaves of plants inoculated with *B. subtilis* showed weak symptoms of disease along the assay. No more progression of *B. cinerea* was observed and colonization of the whole leaves failed by stopping the infection progress (Figure 8C). Therefore, further lesion expansion was drastically reduced in *B. subtilis*-inoculated plants, in contrast to control plants.

## 4. Discussion

### 4.1. Endophytic Microorganisms, an Essential Part of the Plant Microbiome

Many endophytic microorganisms are common species such as *Pseudomonas*, *Burkholderia*, and *Bacillus* [51,52,53,54,55,56]. They produce antimicrobial compounds and have therefore been used as biocontrol agents to control certain pathogens. Moreover, these bacterial genera promote plant growth, as has been described in several studies [18,57,58,59,60,61,62]. Among them, the *Bacillus* genus is considered one of the most abundant in the rhizosphere where it establishes a direct relationship with plants [58]. *Bacillus* spp. have frequently been described as endophytic microbiota of several plant species (cotton, vine, maize, etc.), where they provide disease protection and promote plant growth under certain conditions [63]. *Bacillus* spp. has previously been isolated from maize and even from the direct ancestor of maize, a lowland wild grass known as teosinte, including from roots, leaves, and seeds [48,64,65]. Some isolates from maize plant roots were able to fix nitrogen, produce indole acetic acid, siderophores, and lytic enzymes. In addition, they showed antagonistic effects against *Fusarium verticillioides*, *Colletotrichum graminicola*, *Bipolaris maydis,* and *Cercospora zea*-*maydis* [66], and the ability to break down Aflatoxin B1 [67], and also stimulated plant defences against phytopathogenic fungi [68,69]. In our study, *Bacillus subtilis* strains exhibited many interesting characteristics, including biocontrol in the form of antagonistic activity against *B. cinerea*.

### 4.2. Metabolic Characteristics of Endophytic Microorganisms Are Key in the Development of Host Plants

Endophytic microorganisms are mostly present in farm soil, although they have also been found dormant in seeds. The rhizosphere is therefore the main access route to host plants, and the ability to metabolize many carbon sources enables microorganisms to compete for the exudates secreted by plants in that process [52]. Some plant species can release 40–90% of the carbon fixed in the leaves through their roots [70]. These exudates contain low-molecular-weight (amino acids, organic acids and sugars, among others) and high-molecular-weight compounds (mucilage and proteins) that have a major impact on microbial communities in soil. In the case of exudates from maize plants, they contain mainly water-soluble compounds (79%), of which 64% are carbohydrates (glucose, fructose, and sucrose), 22% amino acids (glutamine, aspartate, and serine), and 14% organic acids (citric and succinic acid) [71]. This study characterized the metabolic capacity of selected strains of *B. subtilis,* which were able to metabolize many carbon sources (Table 3), including L-arabinose, D-ribose, D-glucose, D-fructose, D-manitol, D-sorbitol, esculin, D-maltose, D-sucrose, and D-raffinose. Compounds exuded by plant roots induce chemotactic responses in endophytic bacteria, which give them advantages over other microorganisms when colonizing roots [72].

Other important characteristics of strains of *B. subtilis* that could impact plant growth and protections were also studied: (i) nitrogen fixation, (ii) siderophore production, (iii) indole acetic acid production, (iv) proteolytic activity, (v) amylolytic activity, (vi) biofilm formation, and (vii) phosphate and potassium solubilization. Atmospheric nitrogen (N_2_) is converted into assimilable forms through biological fixation, where diazotrophic bacteria transform it into ammonia using an enzymatic complex called nitrogenase [73]. Cultivation in JMV nitrogen-free medium (Table 4) suggests that they are able to fix atmospheric nitrogen [35]. Although nitrogen fixation by the nitrogenase enzyme is the most studied system, other mechanisms involving endophytic bacteria’s capacity to deliver N_2_ to their host plant could also play a role. Paungfoo-Lonhienne et al., (2010) [74] established that plants are able to incorporate microorganisms into their root systems and then digest and use them as a source of nutrients, a process known as rhizophagy. It has been also demonstrated that some species of herbs, when deprived of nutrients, can extract nitrogen from endophytic bacteria through oxidation by means of reactive oxygen species, a process known as oxidative nitrogen scavenging [75,76].

Another vital factor in plant growth and development is the availability and assimilation of iron. Iron (Fe) is a limiting nutrient that is essential for multiple processes, including the tricarboxylic acid cycle, chlorophyll synthesis, maintenance of chloroplast structure and function, the electron transport chain, oxidative phosphorylation, and photosynthesis [77]. However, despite its abundance in soil, Fe is difficult to extract owing to the insolubility of its oxidized form [78,79]. Some microorganisms are able to produce low-molecular-weight organic compounds known as siderophores that can capture this insoluble iron [77]. *Botrytis cinerea* has the ability to produce several trihydroxamate-type siderophores under limited iron conditions; however, only ferrirodine has been characterized as the predominantly secreted siderophore by this fungus [78]. Bacterial siderophores can provide plants with iron while inhibiting the growth of phytopathogens by competing for this mineral [80]. This metabolic characteristic may be related to two interesting capacities exhibited by our strains. First is its antagonist activity against the pathogen *B. cinerea*, with a high inhibition percentage in co-cultivation confrontations (Figure 2, Table 3). Competition between the two strains may be due to differences in the affinities of their siderophores for iron as described by Hibbing et al. (2010) [81]. Second, *B. subtilis* strains have been shown to promote the growth of roots of maize plants. Siderophore synthesis could be a beneficial characteristic facilitating the assimilation of iron from the substrate to the plant, and hence the improvement root length when compared to maize plants not inoculated with bacteria (Figure 7).

Hormonal regulation during root development is closely related to indole acetic acid (IAA) and abscisic acid (ABA) [82]. IAA is the main auxin found in higher plants and is involved in plant growth and development processes, and in physiological processes such as cell elongation and division, tissue differentiation, phototropism, gravitropism, and defensive responses, and plays a vital role in the formation of xylem and root tissue [83]. IAA produced by plant-growth-promoting bacteria influences the level of auxins in plants, which can lead to a decrease or increase in root length and surface area [84]. The impact of exogenous IAA on plant growth depends on the quantity and sensitivity of the plant tissue to concentrations of this auxin [85]. In our study, the characterized *B. subtilis* strains produced IAA, and this could suggest a causal relationship with enhanced root development in inoculated maize plants as opposed to those that were not inoculated with these strains, although it would be necessary to carry out more studies to ensure these conclusions. Therefore, these strains could be used to sustainably increase crop production [86].

Different studies have evaluated the ability of bacterial proteases to inhibit phytopathogens. *Bacillus* spp. isolated from roots of maize plants, with proteolytic capacity, was related to hyper-parasitic activity and the ability to break down fungi cell walls [66]. *B. subtilis* isolated from the roots of *Curcuma longa* [87] and from strawberry plants [88] exhibited proteolytic activity that was associated with the ability to inhibit the microbial growth of different phytopathogenic fungi, that is, *Aspergillus flavus* [89]. This capacity was correlated to the production of lytic enzymes and proteases from bacterial culture [89]. Our *B. subtilis* strains exhibited proteolytic capacity and high inhibition rates against the fungus *B. cinerea*. According to studies published to date, this proteolytic capacity gives bacteria a chance to fight against phytopathogenic fungi, as has been also shown in the biocontrol capacity in our study.

Therefore, the metabolic characteristics studied in the *B. subtilis* strains isolated in this study endow them with the ability, to some extent, to stimulate plant growth. It can be a direct effect, providing the plant with essential elements for its growth (N_2_ and Fe), or through hormonal stimulation (IAA), or indirectly, by inhibiting the growth of phytopathogenic fungi through the degradation of their cellular structure (proteases) or through competition for substrates (siderophores).

### 4.3. The Co-Culture of B. subtilis vs. B. cinerea Produces Important Morphological Changes and Modifies Botryanes Production

The capacity of some *B. subtilis* isolates to act as biocontrol agents against *B. cinerea* is known [90]. However, no detailed studies on the co-culture of these two microorganisms have been reported. During the co-culture, it was observed that the microorganisms come into contact with no clear zone of inhibition, described previously as ‘contact inhibition’ (Figure 5A) [50]. In *B. subtilis*, at least five different types of cells are affected that are associated with a unique set of phenotypes: motility, surfactin production, matrix production, protease production, and sporulation [91,92]. In addition, these bacteria could move in three different ways under stress conditions, known as swimming, swarming, and sliding [91,93], that is, in response to stimulation with antibiotics triggering a sliding movement response [94]. This type of movement is independent of the flagellum and it depends fundamentally on the secretion of surfactin, the presence of potassium, and the matrix secretion [93,94]. In conjunction with this type of mobility at the edge of the colonies, structures known as “van Gogh bundles” can be formed [92], as we can observe when *B. subtilis* was co-cultivated with *B. cinerea* in this study (Figure 5A).

Botryanes (botrydial and derivatives) are secondary metabolites that play a fundamental role in the pathogenic cycle of *B. cinerea* due to their phytotoxicity. However, their effect is not limited to plants, as the toxin has proven to be cytotoxic and antimicrobial [24,95,96]. A statistically significant increase in fungal toxin production (*p* ˂ 0.05) was observed in the non-interaction zone during co-culture, as opposed to the interaction zone where botryanes concentration did not significantly differ from the control (axenic culture of *B. cinerea*). The increased production in the non-interaction zone coincides with the appearance of hyphae in this same sector, which were observed without any change in their structure (Figure 5B). This could suggest that botryanes production in this zone is *B. cinerea*’s reaction to *B. subtilis*. In addition, considering that filamentous fungi are able to form a network of interconnected hyphae enabling them to act in a coordinated fashion in different parts of a colony [97], the high concentration of botryanes in the non-interaction zone likely indicates that *B. cinerea* could detect antagonist microorganisms in the vicinity and send signals to this zone that trigger the synthesis of botryanes in preparation for an imminent attack. A similar observation was presented during co-culture with *B. amyloliquefaciens* [24]. We studied the interaction zone under a microscope (Figure 5C) and found that *B. subtilis* was on and around the hyphae, which appeared structurally altered, (i.e., granulations and macrosiphonate) (Figure 5C). Therefore, our results would appear to indicate that *B. cinerea* uses these toxins as a defense mechanism against *B. subtilis*.

### 4.4. The Biocontrol Capacity of B. subtilis Could Be Associated with Its Ability to Produce Lipopeptides

It has been established that between 4–5% of the *B. subtilis* genome is devoted to the synthesis of antibiotics. This species has the potential to produce more than two dozen antimicrobial compounds [98]. The biocontrol capacity of many species of the *Bacillus* genus has mainly been associated with the lipopeptide system [99,100]. Lipopeptides found in bacteria associated with plant environments not only inhibit phytopathogens but also play an important role in colonization processes and in inducing resistance responses in the host. Lipopeptides are classified into three families: surfactin, iturin, and fengycin [98]. The lipopeptide measurement of the *B. subtilis* strains isolated in this study showed that they all have the capacity to produce these compounds (Table 4), and these data correlate directly with the mycelial growth inhibition capacity of each of the strains (Table 3). Detection of the genes involved in lipopeptide biosynthesis showed that all strains could synthesize three types of lipopeptides: surfactin, bacillomycin, and the peptide bacilysin (Figure 3B–D). Similar observations were studied by Mora et al., establishing that these three metabolites could play a fundamental role in the competition that takes place in the plant environment [34].

Surfactins plays a fundamental role in the motility of *B. subtilis* given that they are powerful biosurfactants [98]. Due to their nature, surfactins can integrate into the lipid bilayers of cell membranes and interfere with their integrity, and thus act as antimicrobial agents. However, the susceptibility of the membrane to surfactins depends on their sterol content, and this is related to its moderate fungitoxicity [98]. Iturin family compounds (bacillomycin) perform antimicrobial activity by inserting their hydrophobic tails in membranes. Through self-aggregation, these compounds produce pores that generate osmotic disturbance and damage the membrane, thus accounting for the strong antifungal activity of iturins [100]. In addition to acting directly, lipopeptides can trigger plant defenses against phytopathogens and increase their resistance. The work carried out by Farace et al. (2015) [101] showed that grapevine seedlings exposed to surfactins and mycosubtilin (a member of the iturin family) were more resistant to infection caused by *B. cinerea*. These metabolites were able to activate defense genes in the plant, mitigating the damage caused by this fungus [101]. Bacilysin is a non-ribosomal dipeptide (L-Alanine-L-anticapsin), which has antibacterial and antifungal properties [34]. Bacilysin must be transported into the cell before it can act as an antimicrobial agent. This compound then undergoes hydrolysis by intracellular peptidases, causing the release of anticapsin that acts as an inhibitor of the enzyme glucosamine 6-phosphate synthase, thus interfering in cell wall formation [102,103]. The genes detected in *B. subtilis* strains responsible for the synthesis of these three metabolites show that they have an arsenal with great antifungal potential. These genes are probably active during co-culture with *B. cinerea,* and the inhibitory effect is due to their synergistic action. Damage to *B. cinerea* hyphae (Figure 5C) is an example of the effect that these metabolites could be exerting. However, as mentioned above, the proteolytic and siderophore-producing capacity observed in *B. subtilis* strains also could have a potential role of inhibition against *B. cinerea*.

### 4.5. The Non-Specific True Endophyte B. subtilis: Ability to Promote Plant Growth of Zea mays and Biocontrol Agent in Phaseolus vulgaris

Soil fertility in modern agricultural systems is mostly maintained by applying fertilizers. However, only a small portion of this fertilizer is used by plants; between 40 and 70% of the nitrogen, 80–90% of the phosphorus, and 50–70% of the total applied fertilizers are lost in the environment, generating pollution problems [104]. Therefore, one of the main challenges facing current agricultural systems is the production of sustainable and environmentally friendly crops [15]. Many endophytic microorganisms have been classified as plant growth promoters. This promotion is achieved through different mechanisms such as the production of phytohormones, minerals solubilization (phosphate and potassium), nitrogen fixation, and increased tolerance to stress caused by biotic and abiotic factors [9,105]. Therefore, the use of plant growth promoters by means of inoculation is considered an important strategy for sustainable management and for reducing the use of chemical fertilizers, ultimately reducing the environmental impact associated with these compounds [105]. Endophytic bacteria can enter plants through the seeds and/or reproductive organs. They can by be passed on from one generation to the next. They can also enter plants through the roots and stomata with the aid of sap-feeding and pollinating insects [52]. Of these different routes of entry for endophytic bacteria, roots are still considered the most important and the rhizosphere the main source of these microorganisms. Plants’ growth stage also plays a fundamental role in the entry of endophytic bacteria [55].

A recent artificial endophytic microorganism inoculation technology applied to agriculture consists of the encapsulation of microorganisms in a polymeric matrix. In recent years, this technique has had a great impact on the fermentation industry [106]. Encapsulating bacteria for use in agriculture is intended to (i) provide a temporary refuge for the encapsulated strain against biotic and abiotic soil factors and (ii) gradually release the desired strain to colonize plant roots [106].

In this work, *B. subtilis* cells were encapsulated in alginate beads and remained viable throughout the inoculation process (Figure 6A,B). Alginate is the most used polymer to encapsulate plant-growth-promoting microorganisms such as *Azospirillum brasilense*, *Pseudomonas fluorescens*, *Bacillus pumilus*, *Bacillus subtilis*, *Pseudomonas* spp., and others [107]. Alginate is preferred by bacterial cultures because it is a natural product, non-toxic, biodegradable, and cheap. Bead preparation is straightforward, requires simple instruments, and provides for the gradual release of microorganisms in the soil [107]. Colonies of *B. subtilis* were re-isolated from inoculated plants: *Z. mays* and *P. vulgaris*. In addition, positive PCR amplification using total DNA samples from inoculated plant confirmed the presence of *B. subtilis* inside inoculated plants and its absence in control plants. This suggests that the inoculant was released from the alginate beads, penetrated the plant, and established a symbiotic relationship; in turn, a beneficial impact on plant development was observed. Moreover, these results show that endophytic *B. subtilis* could be non-specific to hosts, establishing endophytic relationships with two species of different types of plants, *Z. mays* (family Poaceae) and *P. vulgaris* (family Fabaceae). For a microorganism to be considered a true endophyte, not only must it be isolated from previously disinfected plant tissue, there must also be microscopic evidence of its presence within plant tissue [108]. Microorganisms that meet only the first criterion are classified as putative endophytes. However, the joint use of molecular techniques and classical microbiology (detecting bacterial DNA in plant tissue and re-isolating it from sterilized tissue) have demonstrated that the *Bacillus subtilis* 6Sm strain is able to enter the tissue of maize and bean plants and establish itself as a true endophyte. A similar strategy was carried out to demonstrate the ability of *Erwinia* and *Paenibacillus* as true endophytes from wheat [109].

As the results show, the *B. subtilis* 6Sm strain encapsulated in alginate beads stimulated root growth in maize plants, the difference with the control being statistically significant (*p* ˂ 0.05) (Table 5). This ability to enhance root growth could be related to the production of IAA (Table 4), as mentioned above, and as indicated in publication regarding melon and tomato plants by Zhao et al. (2011) and Walia et al., (2014), respectively [110,111]. To study the role of *B. subtilis* as biocontrol agent against *B. cinerea* during in vivo infections, *P. vulgaris* was chosen as a classical sensible host of the phytopathogen [30]. Results show that infection was delayed, and leaves were not completely colonized by *B. cinerea* along the pathogenicity assay. These results could suggest that the presence of *B. subtilis* inside the bean plants would give a certain degree of protection against the infection developed by *B. cinerea*.

Given its ability to inhibit *B. cinerea*, produce siderophores, produce IAA, and produce lipopeptides and antimicrobial peptides, *B. subtilis* 6Sm has great potential for use as a biocontrol agent and plant-growth-promoting bacterium. All these assays should be scaled up and carried out in cultivation fields with real conditions of growth and infection in the field.

## 5. Conclusions

In recent decades, the search for more sustainable agricultural systems has increased interest in new microorganisms with the capacity to promote plant growth. Among these microorganisms, endophytes have become extremely relevant owing to their close relationship with host plants, in addition to sharing the same ecological niche as some phytopathogens, increasing their value as candidates for biocontrol. In this work, five strains of *B. subtilis* were isolated from maize plants grown in Colombia. The strain identified as *B. subtilis* 6Sm exhibited characteristics earning its classification as a plant-growth-promoting bacterium. Furthermore, this strain was successful in inhibiting *B. cinerea* growth in vitro, suggesting its suitability as a biocontrol agent. As Colombia is considered the second most biodiverse country in the world, this could be the beginning of an ongoing search for endophytic microorganisms from plants with great biotechnological potential for the agri-food industry.

## Figures and Tables

**Table 1:** Strains used in this study.

Strains	Species	Origin of Isolate	GenBank Acc. N.	References
**WT:B05.10**	*Botrytis cinerea*	*Vitis vinifera*	ASM14353v4	[25]
**9Ca**	*Pseudomonas aeruginosa*	*Zea mays*	--	Laboratory collection
**2S**	*Bacillus subtilis*	*Zea mays*	MW204831	This study
**5Cs**	*Bacillus subtilis*	*Zea mays*	MW204832	This study
**5Cm**	*Bacillus subtilis*	*Zea mays*	MW204833	This study
**6Ss**	*Bacillus subtilis*	*Zea mays*	MW204834	This study
**6Sm**	*Bacillus subtilis*	*Zea mays*	MW204835	This study

**Table 2:** Primers used in this study.

Primer	Sequence (5′ → 3′)	Product Size (bp)	Reference	Used for
16SF	AGAGTTTGATCCTGGCTCAG	1500	[31]	16S-rRNA partial amplification
16SR	TACGGCTACCTTGTTACGA	1500	[31]	16S-rRNA partial amplification
Bac_FWd	AGCAGTGGGGAATATTGGAC	700	This study	16S-rRNA partial amplification
Bac_Rev1	TCTAATCCTGTTTGCTCCCC	700	This study	16S-rRNA partial amplification
Bsub5F	AAGTCGAGCGGACAGATGG	600	[33]	Species-specific primers for *B. subtilis* identification
Bsub3R	CCAGTTTCCAATGACCCTCCCC	600	[33]	Species-specific primers for *B. subtilis* identification
ITUCF	GGCTGCTGCAGATGCTTTAT	423	[34]	Detection of *ituC* gene (Iturin)
ITUCR	TCGCAGATAATCGCAGTGAG	423	[34]	Detection of *ituC* gene (Iturin)
FENDF	GGCCCGTTCTCTAAATCCAT	270	[34]	Detection of *fenD* gene (Fengycin)
FENDR	GTCATGCTGACGAGAGCAAA	270	[34]	Detection of *fenD* gene (Fengycin)
BACF	CAGCTCATGGGAATGCTTTT	500	[34]	Detection of *bacA* gene (Bacylisin)
BACR	CTCGGTCCTGAAGGGACAAG	500	[34]	Detection of *bacA* gene (Bacylisin)
SRFAF	TCGGGACAGGAAGACATCAT	200	[34]	Detection of *sfrAA* gene (Surfactin)
SRFAR	CCACTCAAACGGATAATCCTGA	200	[34]	Detection of *sfrAA* gene (Surfactin)
SPASF	GGTTTGTTGGATGGAGCTGT	375	[34]	Detection of *spaS* gene (Subtilin)
SPASR	GCAAGGAGTCAGAGCAAGGT	375	[34]	Detection of *spaS* gene (Subtilin)
BMYBF	GAATCCCGTTGTTCTCCAAA	370	[34]	Detection of *bmyB* gene (Bacillomycin)
BMYBR	GCGGGTATTGAATGCTTGTT	370	[34]	Detection of *bmyB* gene (Bacillomycin)

**Table 3:** Cell morphology and biochemical characteristics of carbon metabolisms and enzymatic activity of the five selected bacteria.

Title	*B. subtilis* Strains				
Characteristics	2S	5Cs	5Cm	6Ss	6Sm
% Inhibition *B. cinerea*	46	53	48	42	54
Shape	Rod	Rod	Rod	Rod	Rod
Gram	+	+	+	+	+
Oxidase	+	+	+	+	+
Catalase	+	+	+	+	+
Motility	+	+	+	+	+
Glycerol	+	+	+	+	+
Erythrose	−	−	−	−	−
D-Arabinose	−	−	−	−	−
L-Arabinose	+	+	+	+	+
D-Ribose	+	+	+	+	+
D-Galactose	−	−	−	−	−
D-Glucose	+	+	+	+	+
D-Fructose	+	+	+	+	+
D-Mannitol	+	+	+	+	+
D-Sorbitol	+	+	+	+	+
Esculin	+	+	+	+	+
D-Maltose	+	+	+	+	+
D-Lactose	−	−	−	−	−
D-Sucrose	+	+	+	+	+
D-Raffinose	+	+	+	+	+
Starch	+	+	+	+	+
Glycogen	+	+	+	+	+

**Table 4:** Metabolic characterization of endophytic bacteria strains as relates to the promotion of plant growth.

Title	*B. subtilis* Isolates				
Characteristics	2S	5Cs	5Cm	6Ss	6Sm
IAA (μg·mL^−1^)	1.6	3.7	2.6	1.4	2.2
Phosphate solubilization	-	-	-	-	-
Potassium solubilization	-	-	-	-	-
Growth in nitrogen-free medium	+	+	+	+	+
Proteolytic activity	+	+	+	+	+
Amylolytic activity	+	+	+	+	+
Siderophore detection	+	+	+	+	+
Biofilm formation	-	-	-	-	-
Lipopeptide production (mg·mL^−1^)	1.00	1.05	0.94	0.76	1.24

**Table 5:** Plant-growth-promoting characteristics.

Strain	Wet Weight (g)	Number of Leaves	Stem Length (cm)	Root Length * (cm)
*No bacteria*	6.7 ± 1.06	6 ± 0.46	14.9 ± 1.55	47.9 ± 1.25 *
*B. subtilis* 6Sm	7.0 ± 0.52	7 ± 0.46	15.6 ± 0.74	60.5 ± 0.80 *

* Statistically significant difference (*p* ˂ 0.05).

## Supplementary Materials

The following are available online at https://www.mdpi.com/article/10.3390/biology10060492/s1, File S1: Climate of The Department of Atlántico.
