# Supplementary material for: Endophytic Bacteria Bacillus subtilis, Isolated from Zea mays, as Potential Biocontrol Agent against Botrytis cinerea
Source: Biology (Basel). 2021 Jun 1;10(6):492. doi: 10.3390/biology10060492 (PMC8229056; doi:10.3390/biology10060492)
Supplement: Supplementary file 1 [file biology-10-00492-s001.zip › biology-1190722-supplementary.pdf]

Article

Endophytic bacteria *Bacillus subtilis,* isolated from *Zea mays,* as potential biocontrol agent against *Botrytis cinerea*

Hernando José Bolivar-Anillo[2,3], Victoria E. González-Rodríguez[1], Jesús M. Cantoral[1], Darío García-Sánchez[1], Isidro G. Collado[2*] and Carlos Garrido[1*]

SUPPLEMENTARY MATERIAL:

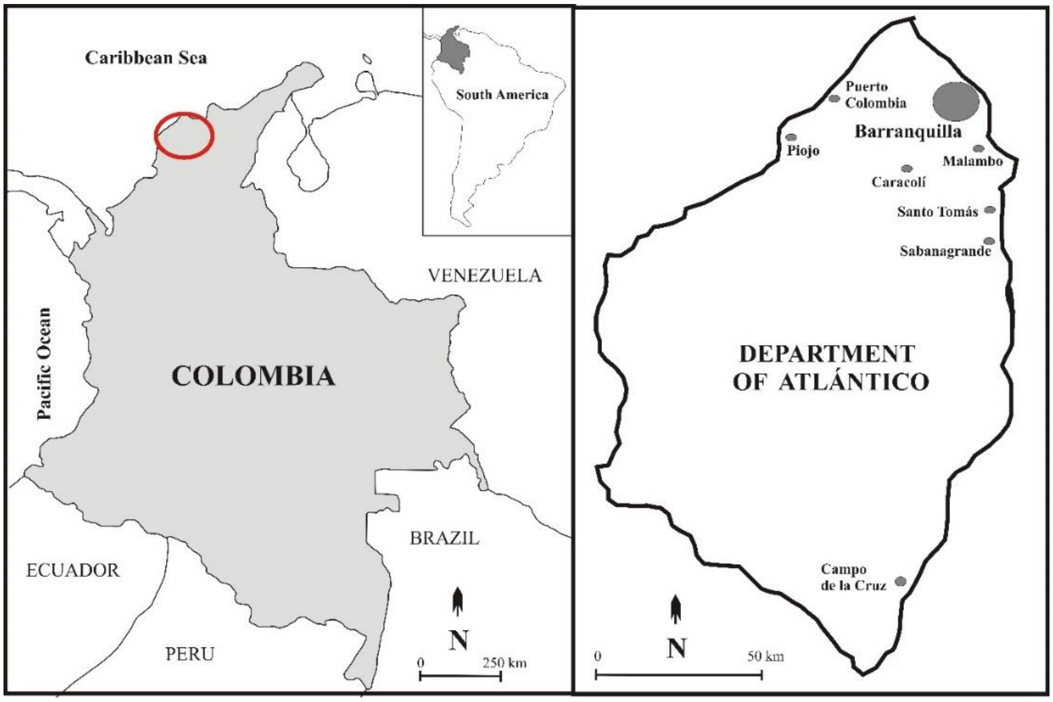

Figure 1. Department of Atlántico region in Colombia. On the left the Colombia map with a red mark of sample region. On the right the Department of Atlántico with the name sampling areas/towns.

CLIMATE OF THE DEPARTMENT OF ATLÁNTICO

The total annual rainfall does not exceed 1500 mm and in some sectors of the northeast and south-west of the department, the rains oscillate between 500 and 1000 mm. The interannual regimen is bimodal. The main rainy season runs from September to November and in the first semester there is a short season of increased rainfall, mainly in the month of May. The dry seasons occur between December and April, the main one, and a second, less intense, mid-year, in the months of June, July and August. The number of rainy days during the year ranges between 50 and 100, in most of the department. In some isolated spots, it rains for less than 50 days a year.

TEMPERATURE. The average temperature in the department exceeds 26°C. In southern sectors more than 28°C are recorded on average during the year.

CLIMATE CLASSIFICATION As a consequence of the prevailing rainfall and thermal regime in the department, the climates recorded are hot, arid and semi-arid.

Reference:

Instituto de Hidrología, Meteorología y Estudios Ambientales (IDEAM). Atlas Climatológico de Colombia. Available online:

http://atlas.ideam.gov.co/visorAtlasClimatologico.html (accessed on 11 May 2021).

Location	Soil characteristic
Puerto Colombia	Shallow and deep, well to moderately drained, fine and moderately coarse textures, very strongly acidic to neutral and very high base saturation. Presence of gypsum, salts and/or sodium in the deepest horizons.
Malambo	Moderately deep and shallow, well to poorly drained, fine and moderately coarse textures, acid on the surface and alkaline in depth and high base saturation.
Caracolí	Deep and shallow, well and poorly drained, coarse and fine textures, slightly acidic to slightly alkaline, and high base saturation.
Santo Tomas<br>Sabana Grande<br>Campo de la Cruz	Moderately deep and superficial, imperfect or poorly drained, fine and moderately coarse textures, acid on the surface and alkaline in depth and very high base saturation.
Piojo	Moderately deep, well drained, fine, moderately fine, and moderately coarse textures, slightly acidic to neutral, and high base saturation.

Reference:

Instituto Geográfico Agustín Codazzi Subdirección de Agrología. Estudio General de Suelos y Zonificación de Tierras Departamento del Atlántico. Bogotá: Imprenta Nacional de Colombia. 2008.

Original Text in Spanish:

CLIMA DEL DEPARTAMENTO DEL ATLANTICO

Las precipitaciones totales anuales no sobrepasan los 1500 mm y en algunos sectores del noreste y del suroccidente del departamento, las lluvias oscilan entre 500 y 1000 mm. El régimen intranual es de tipo bimodal. La principal temporada de lluvias se extiende de septiembre a noviembre y en el primer semestre se registra una corta temporada de aumento de lluvias principalmente en el mes de mayo. Las temporadas secas ocurren entre diciembre y abril, la principal, y una segunda, de menor intensidad, mediados de año, en los meses de junio, julio y agosto. El número de días lluviosos durante el año oscila entre 50 y 100, en la mayor parte del departamento. En algunos puntos aislados, llueve durante menos de 50 días al año.

TEMPERATURA. La temperatura media en el departamento supera los 26°C. En sectores del sur se registran más de 28°C en promedio durante el año.

CLASIFICACIÓN CLIMÁTICA Como consecuencia del régimen pluvial y térmico dominante en el departamento, los climas que se registran son de tipo cálido árido y semiárido.

Referencia:

Instituto de Hidrología, Meteorología y Estudios Ambientales (IDEAM). Atlas Climatológico de Colombia. Available online: http://atlas.ideam.gov.co/visorAtlasClimatologico.html (accessed on 11 May 2021).

**Municipio**	**Característica del suelo**
Puerto Colombia	Superficiales y profundos, bien a moderadamente drenados, texturas finas y moderadamente gruesas, muy fuertemente ácidos a neutros y saturación de bases muy altas. Presencia de yeso, sales y/o sodio en los horizontes más profundos.
Malambo	Moderadamente profundos y superficiales, bien a pobremente drenados, texturas finas y moderadamente gruesas, ácidos en superficie y alcalinos en profundidad y saturación de base alta.
Caracolí	Profundos y superficiales, bien y pobremente drenados, texturas gruesas y finas, ligeramente ácidos a ligeramente alcalinos y saturación de base alta.
Santo Tomas<br>Sabana Grande<br>Campo de la Cruz	Moderadamente profundos y superficiales, imperfecta o pobremente drenados, texturas finas y moderadamente gruesas, ácidos en superficie y alcalinos en profundidad y saturación de bases muy altos.

Piojo	Moderadamente profundos, bien drenados, texturas finas, moderadamente finas y moderadamente gruesas, ligeramente ácidos a neutros y saturación de bases altas.

Referencia:

Instituto Geográfico Agustín Codazzi Subdirección de Agrología. Estudio General de Suelos y Zonificación de Tierras Departamento del Atlántico. Bogotá: Imprenta Nacional de Colombia. 2008.
